# A One-Pot CRISPR/Cas12a-Based Platform for Contamination-Free Nucleic Acid Amplification Detection

**DOI:** 10.3390/bios16030170

**Published:** 2026-03-19

**Authors:** Wei Tantai, Qinfeng Xu, Wenjuan Zhang, Yanni Li, Hao Liu

**Affiliations:** School of Food Science and Engineering, National R & D Center for Goat Dairy Products Processing Technology, Shaanxi University of Science and Technology, Xi’an 710021, China; bs200411006@sust.edu.cn (W.T.); 1804013@sust.edu.cn (W.Z.); 200411024@sust.edu.cn (Y.L.); bs220411004@sust.edu.cn (H.L.)

**Keywords:** Cas12a, contamination-free, visual nucleic acid detection, one-pot

## Abstract

CRISPR-Cas12a enables rapid and specific detection of PCR/LAMP (loop-mediated isothermal amplification) reaction products; however, this approach often requires open-tube manipulation, rendering it prone to cross-contamination. Here, we developed a novel one-pot reaction system that eliminated carryover contamination and facilitated endpoint detection using a CRISPR/Cas12a-based system. We leveraged the dependence of the CRISPR-Cas12a cleavage system on the protospacer-adjacent motif (PAM) to design PCR/LAMP primers that incorporated the PAM site (TTT) into amplified DNA. Pre-incubation of Cas12a with crRNA1 and crRNA2 using PCR/LAMP resulted in efficient cleavage of cross-contaminating DNA, while the target gene remained intact due to the lack of PAM sites. Furthermore, a Cas12a-detection complex (comprising Cas12a, crRNA3, trehalose, and the ssDNA probe) pre-stored on the lid was introduced to mix with the PCR/LAMP amplicons, which triggered the non-specific cleavage of fluorescent probes for direct visual detection under a blue LED instrument. This method effectively degraded up to 10^6^ copies of carryover contaminants within one hour, demonstrating the potential of one-pot detection methods in complex samples.

## 1. Introduction

Nucleic acid-based assays are rapidly evolving, providing opportunities for more accurate diagnoses with additional insights [1]. For example, PCR/LAMP was employed to detect SARS-CoV-2 RNA, providing rapid nucleic acid test results for public health and prevention during the COVID-19 pandemic [2]. However, nucleic acid amplification techniques such as PCR and LAMP are prone to false-positive results due to contamination, which limits their application in timely diagnosis [3,4]. Their high sensitivity is often compromised by residual contamination arising from the operating environment, in which aerosolized products from previous amplification reactions may act as new templates for re-reaction, resulting in false-positive outcomes [5]. To prevent such contamination, operators often rely on spatial isolation. One-tube manipulation and chemical treatment strategies (e.g., UV irradiation, hydrolytic enzymes, and uracil-N-glycosylase treatment) can also help mitigate contamination risk [6,7,8,9]. Furthermore, some reports indicated that once contamination from nucleic acid amplification occurs, it is difficult to completely reduce or eliminate [4,10].

The CRISPR/Cas technique has become a promising tool for addressing this issue.

Fundamentally, CRISPR/Cas systems function as highly precise, RNA-guided endonucleases that rely on a specific guide RNA (gRNA) to find a complementary target sequence. Crucially, successful recognition and cleavage strictly require a protospacer-adjacent motif (PAM) to activate DNA cleavage activity [11,12]. By applying this concept, early studies explored Cas9-based contaminant-free strategies. For example, Bao et al. leveraged the precise searching and cleavage performance of the CRISPR/Cas system to develop a Cas9-based contaminant-free LAMP strategy (CUT-LAMP) that allowed for the removal of contaminants after they were present in the environment [13]. Similarly, Lin et al. proposed the use of a Cas9 erasing strategy (CASLFA) for RT-PCR to achieve contamination-free RNA detection [14]. Because the cleavage site of Cas9 is only 3 nt relative to the PAM, this small gap constrains the placement of PAM insertion, resulting in the possibility that the post-cut products may support the extension of primers (Figure S1). Furthermore, the subsequent macroscopic confirmation of amplification results (e.g., fluorescent dye) may lead to further cross-contamination in CUT-LAMP.

Recently, Cas12 has emerged as a powerful alternative to Cas9 due to its excellent sequence-specific recognition and cleavage of target genes. Cas12, under the guidance of its CRISPR RNA (crRNA), can specifically cleave the target DNA (termed *cis*-cleavage) and is subsequently activated to nonspecifically cleave surrounding single-stranded DNA reporters (termed *trans*-cleavage). Combined with its T-rich PAM recognition and short guide RNAs, these properties enable rapid and accurate detection of specific targets [15,16]. Wang et al. coupled recombinase polymerase amplification (RPA) with a Cas12a reaction to reduce the risk of cross-contamination using a one-pot assay, but this operation alone did not completely eliminate pre-existing cross-contamination [17]. Currently, many research groups have proposed a combined strategy using UDG to remove contaminants while using Cas12 to detect the target [18,19,20]. However, this strategy requires the use of two proteins, which increases the test complexity. Meanwhile, the Cas12 protein was primarily used to specifically recognize and detect target DNA rather than contamination in this strategy. Therefore, there are clear advantages to developing a one-pot CRISPR/Cas12-based platform to avoid carryover contamination.

To address this unmet need, we developed a novel one-pot reaction system capable of eliminating carryover contamination and providing endpoint detection using a CRISPR/Cas12a-based system. The core of our approach hinged on the dependence of the CRISPR/Cas12a cleavage system on the PAM site, which allowed the design of PCR/LAMP primers that contained the TTT base sequence. Additionally, two types of crRNAs were used (crRNA1 and crRNA2 for contamination elimination and crRNA3 for detection). In our system, the Cas12a-based contamination-free detection and visualization reactions were performed in separate areas within a single tube to prevent interaction. Specifically, the reagents for amplification and contamination elimination were placed at the bottom, while the Cas12a-detection complex (comprising Cas12a, crRNA3, trehalose, and the ssDNA probe) was pre-stored on the tube lid. Additionally, we found that certain sugar solutions stabilized or activated Cas12a enzyme activity at elevated temperatures, effectively rendering the enzyme heat-resistant.

## 2. Materials and Methods

### 2.1. Materials

EnGen^®^ LbaCas12a (M0653s), 10× NEBuffer r2.1, antarctic thermolabile UDG (M0372S), and the LAMP reagents, including Bst 2.0 WarmStart DNA polymerase (M0538S), MgSO_4_ (B1003S), dNTPs (N0447S), and 10× LAMP amplification buffer (2 mM MgSO_4_, 10 mM (NH_4_)_2_SO_4_, 50 mM KCl, 20 mM Tris-HCl, 0.1% Tween-20), were purchased from New England Biolabs (Ipswich, MA, USA). A 2× Taq PCR reaction mix was acquired from Tiangen Biotech (Beijing, China). Evagreen (20×) was obtained from Biotium (Hayward, CA, USA). A recombinant RNase inhibitor, diethylpyrocarbonate (DEPC)-treated water, and pullulan were acquired from Sangon Biotechnology (Shanghai, China). D-(+)-Trehalose dihydrate, sucrose, and sorbitol were obtained from Sigma (St. Louis, MO, USA). All PCR and LAMP amplifications were performed in a MyGo Pro real-time PCR instrument (IT-IS Life Science Ltd., Middlesbrough, UK). The gel imaging and fluorescence assay were performed on a blue light LED transmission instrument, SMOBIO B-BOX (Model VE0100, Taiwan, China). All primers and ssDNA probes were synthesized by Sangon Biotechnology (Shanghai, China). The gRNA oligonucleotides were synthesized and HPLC-purified by TaKaRa Bio, Inc. (Dalian, China). Data analysis and graphing were performed using OriginPro 9 software (OriginLab Corporation, Northampton, MA, USA).

### 2.2. PCR Reaction and Melting Curve Analysis

(a) For a typical PCR reaction, a 10 μL reaction solution was prepared by mixing 5.0 μL of PCR mix (2×) with 0.4 μM forward primer (PAM-FP), 0.4 μM reverse primer (PAM-RP), the DNA binding dye EvaGreen (1×), and 1.0 μL of the *Listeria monocytogenes* (*L. monocytogenes*) template. The standard PCR was performed using the MyGo Pro real-time PCR instrument, initiating at 95 °C for 5 min, followed by 30 cycles of 95 °C for 15 s, 57 °C for 15 s, and 68 °C for 30 s.
(b) The PAM-PCR experiment was performed with a 10 μL reaction volume comprising 5.0 μL of PCR mix (2×), 0.4 μM PAM-FP, 0.4 μM PAM-RP, the DNA binding dye EvaGreen (1×), 0.1 μM Cas12a, 0.05 μM crRNA1 and crRNA2, and 10 U of recombinant RNase inhibitor. LbCas12a-crRNA1 & 2 complexes were pre-assembled by mixing and incubating for 5 min at 37 °C. Then, the target DNA of *L. monocytogenes* (1 μL) was incorporated into the mixture. The PAM-PCR was initiated on the MyGo Pro real-time PCR device at 37 °C for 10 min, and the remaining steps were the same as the PCR procedure described above.
(c) The UDG assay in the PCR system was performed with a 10 μL reaction volume comprising 0.02 U/μL of UDG, 0.75 mM dUTP, and 3 mM MgCl_2_, as well as other reagents used in PCR. The reaction was performed under the same conditions as the PAM-PCR described above.
(d) To achieve one-pot visual detection, a 10 μL mixture comprising PCR mix (1×) with 0.4 μM PAM-FP, 0.4 μM PAM-RP, 0.1 μM Cas12a, 0.05 μM crRNA1 and crRNA2, and 10 U of recombinant RNase inhibitor was introduced to the lower section of the reaction tube. Additionally, a 6 μL liquid droplet of the Cas12a-detection complex, containing 0.6 M trehalose, 0.1 μM Cas12a, 1× NEBuffer 2.1, 100 nM fluorophore–quencher–labeled ssDNA reporters (ssDNA probes), and 0.1 μM crRNA3, was placed on the cap of the reaction tube. The reagent at the bottom was covered with 5 μL of mineral oil to limit heat transfer. Unless otherwise indicated, all steps were the same as the PAM-PCR procedure described above. Then, the Cas12a cleavage system, which was placed in advance on the tube cap, was mixed with the amplification product at the bottom of the tube by manually turning and shaking the tube. The reaction was permitted to continue for 10 min at 37 °C. Finally, a fluorescence change was observed using a blue light transmission instrument. To ensure the reliability of our method, all experiments were performed in triplicate.

For the PCR assay, the sequences of specific primers, crRNAs, and the target gene are provided in Tables S1, S3 and S4, respectively.

### 2.3. LAMP Reaction and Melting Curve Analysis

(a) For a typical LAMP reaction, a 10 μL reaction solution was prepared by adding 1× LAMP amplification buffer, 0.8 M betaine, 0.1 μM forward primer (F3), 0.1 μM backward primer (B3), 0.8 μM forward inner primer (FIP), 0.8 μM backward inner primer (BIP), 4.5 mM MgSO_4_, 3.2 U of Bst 2.0 WarmStart DNA polymerase, 1.4 mM dNTP mix, 1.0 μL of the template (*L. monocytogenes*), and 0.25 μL of EvaGreen (20×). LAMP was performed on the PCR instrument (MyGo Pro) at 65 °C for 45 min.
(b) The PAM-LAMP experiments were executed with a 10 μL reaction volume comprising 1× LAMP amplification buffer, 0.8 M betaine, 0.1 μM F3, 0.1 μM B3, 0.8 μM FIP, 0.8 μM BIP, 3.2 U of Bst 2.0 WarmStart DNA polymerase, 1.4 mM dNTP mix, 0.1 μM Cas12a, 0.25 μL of EvaGreen (20×), 0.05 μM crRNA1 and crRNA2, and 10 U of recombinant RNase inhibitor. LbCas12a-crRNA1 & 2 complexes were pre-assembled by mixing and incubating for 5 min at 37 °C. Then, the target DNA of *L. monocytogenes* (1 μL) was incorporated into the solution mixture. The PAM-LAMP reactions were tested at 37 °C for 10 min, followed by a 45 min incubation at 65 °C.
(c) To achieve one-pot visual detection, a 10 μL reaction solution comprising 1× LAMP mix with 0.8 M betaine, 0.1 μM F3 and B3, 0.8 μM FIP and BIP, 3.2 U of Bst 2.0 WarmStart DNA polymerase, 1.4 mM dNTP mix, 0.1 μM Cas12a, 0.05 μM crRNA1 and crRNA2, and 10 U of recombinant RNase inhibitor was introduced to the lower section of the reaction tube. Additionally, a 6 μL liquid droplet of the Cas12a-detection complex, which contained 0.6 M trehalose, 0.1 μM Cas12a, 1× NEBuffer 2.1, 100 nM ssDNA probes, and 0.1 μM crRNA3, was placed on the cap of the reaction tube. The mixture at the bottom of the tube was covered with 5 μL of mineral oil to limit heat transfer. Following a 10 min incubation at 37 °C and a subsequent 45 min incubation at 62 °C, the Cas12a system was combined with the amplification product by manually inverting and shaking the tube. The reaction was then allowed to proceed for 10 min at 37 °C. Finally, the fluorescence change was observed using a blue light transmission instrument. To ensure the reliability of our method, all experiments were performed in triplicate.

For the LAMP assay, the sequences of specific primers, crRNAs, and the target gene are provided in Tables S2, S3 and S4, respectively.

### 2.4. Cas12a Activity Assay

The Cas12a assay was carried out in a reaction mixture of 10 μL, incorporating 0.1 μM Cas12a, 1× NEBuffer 2.1 (10 mM MgCl_2_, 100 μg/mL BSA, 10 mM Tris-HCl, 50 mM NaCl, pH 7.9), 0.1 μM probe, and 0.1 μM of the appropriate crRNAs. Then, the target DNA (1 μL) was introduced, and the reaction was tested at 37 °C for 30 min.

### 2.5. Analysis of the Effects of Sugar Solutions on Cas12a Thermostability

The thermostability of Cas12a in the presence of various sugars (pullulan, sucrose, trehalose, and sorbitol), including a negative control condition without added sugar, was evaluated by measuring its residual catalytic activity after thermal incubation. Specifically, the Cas12a-crRNA complex was incubated in NEBuffer 2.1 at 37 °C, 45 °C, 54 °C, 60 °C, and 70 °C for 10 min with pullulan, sucrose, trehalose, or sorbitol or without sugar. Then, 100 nM probes and 1 μL of DNA were introduced, and the solutions were incubated at 37 °C for 30 min to activate the *trans*-cleavage assay. The thermostability was evaluated based on the fluorescence intensity, which reflects the functional integrity of the enzyme after heat treatment. Additionally, 37 °C and 54 °C were also selected as fixed temperatures for testing various concentrations of trehalose (0, 0.3, 0.5, 0.6, 0.7, and 0.9 M) to determine the optimal concentration for maintaining enzyme stability.

## 3. Results and Discussion

### 3.1. The Principle and Feasibility of the Established Method

**Figure 1:**
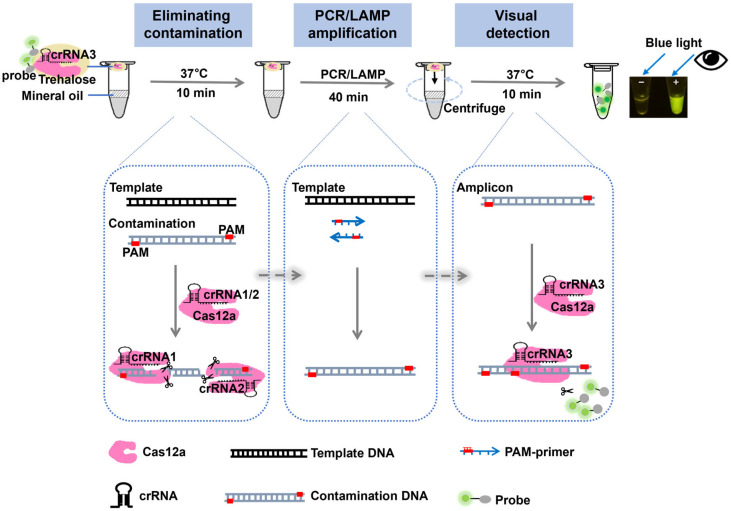
Schematic overview of the one-pot Cas12a-based platform for contamination-free visual nucleic acid detection, including Cas12a-mediated degradation of contaminating amplicons in Step 1, PCR/LAMP amplification in Step 2, and Cas12a *trans*-cleavage-based visual detection in Step.

To minimize carryover contamination caused by tube opening, we developed a one-pot approach that integrated template amplification with CRISPR-Cas12a-based detection. The principle of this approach is shown in Figure 1. First, we used the dependence of the CRISPR-Cas12a cleavage system on the PAM site to design PCR/LAMP primers, ensuring that the final amplification products included the requisite Cas12a PAM recognition sites (TTT). Prior to the next amplification reaction, the reaction mixture was treated with the Cas12a enzyme at 37 °C for only 10 min. The Cas12a-crRNA1 & 2 complexes specifically recognized contaminating amplicons carrying the introduced PAM sites, thereby triggering *cis*-cleavage and enabling rapid degradation of carryover contaminants. In contrast, the target DNA remained intact owing to the absence of the corresponding PAM site. Subsequently, the inherent high-temperature PCR/LAMP cycles inactivated the Cas12a enzyme at the bottom of the tube and enriched the amplified product. To achieve closed-tube visual detection, a separate Cas12a-detection complex (comprising Cas12a, crRNA3, trehalose, and a fluorophore-quencher labeled ssDNA probe) was pre-stored on the lid. After amplification, the tube was centrifuged or shaken to thoroughly mix the reaction system. The resulting amplicons then activated the *trans*-cleavage activity of the Cas12a-crRNA3 complex, leading to nonspecific cleavage of the ssDNA probe. Finally, the fluorescence signal was visualized using a blue light transmission instrument.

As a proof-of-concept approach to testing this assay platform, PCR amplification was performed using both ordinary primers (FP and RP) and primers carrying PAM sites (PAM-FP and PAM-RP). As shown in Figure S3a, amplification curves similar to those obtained with the ordinary primers were observed for the PAM primers, indicating that the PAM primers had no adverse effect on PCR amplification. Furthermore, the amplification products from different primers were verified and analyzed by gel electrophoresis (Figure S3b).

Nevertheless, after recognition and cleavage of contaminating amplicons, activated Cas12a may also exert nonspecific *trans*-cleavage activity toward ssDNA in the reaction system, including primers, which affected subsequent target enrichment [21]. Therefore, the reaction conditions were further optimized to minimize undesired *trans*-cleavage while preserving efficient *cis*-cleavage for contaminant elimination.

### 3.2. Establishment of a Contamination-Free Single-Tube PCR Assay

**Figure 2:**
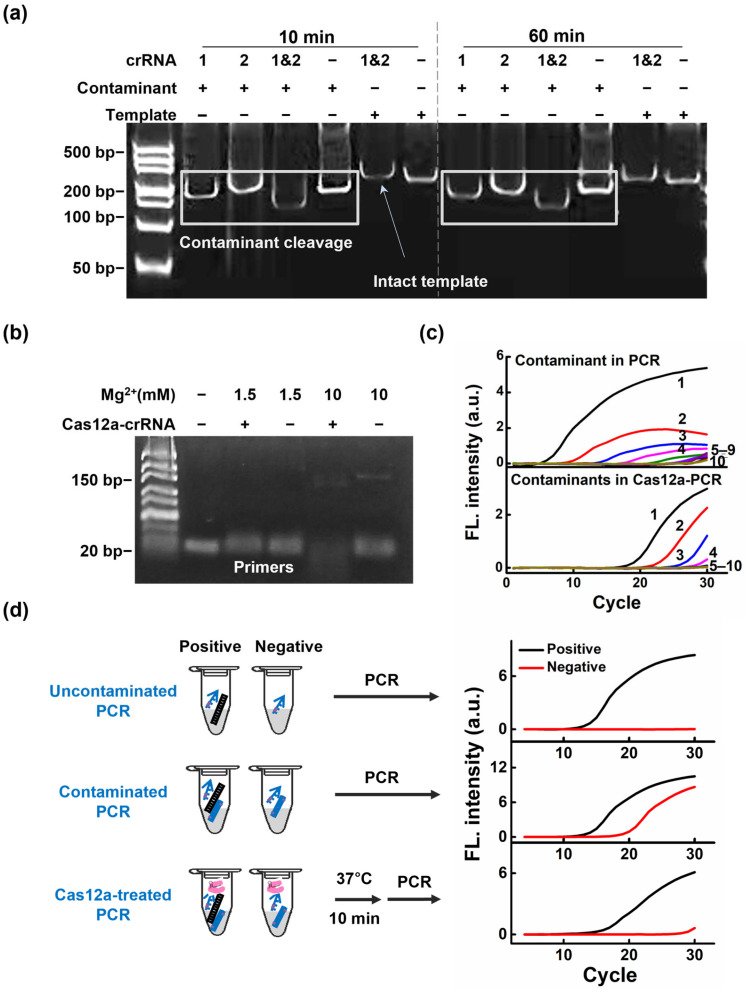
Establishment of a contamination-free single-tube PCR assay. (**a**) Gel analysis of Cas12a-mediated contaminant cleavage after 10 min and 60 min pre-incubation. Contaminant amplicons containing the engineered PAM site were treated with Cas12a-crRNA1, Cas12a-crRNA2, dual Cas12a-crRNAs (1 & 2), or no crRNA, while the authentic target template lacking the corresponding PAM site was analyzed in parallel. Boxed regions indicate the contaminant cleavage assay, and the arrow marks the intact template band. (**b**) Gel analysis of primer stability at different Mg^2+^ concentrations in the Cas12a-crRNA system. (**c**) Real-time amplification analysis of carryover contaminants in the presence and absence of Cas12a. Cas12a-free reactions exhibited amplification with as few as 10^1^ copies of carryover contaminant (upper panel). In contrast, Cas12a treatment effectively eliminated this amplification (lower panel). Labels 1–9 indicate contaminant concentrations (10^9^ copies to 10^1^ copies, respectively, and 10 indicates NTC (no DNA). (**d**) The practical application of Cas12a for digesting false-positive outcomes caused by PCR carryover contamination. Conventional PCR results without cross-contamination (upper panel). False-positive results due to contamination from previous reactions as a template (middle panel). Results of eliminating false-positive amplification by adding Cas12a to cleave contaminants containing PAM sites (lower panel). n the schematic, black and blue dsDNA represent templates and contaminants, respectively; blue arrows represent primers; pink symbols represent Cas12a. In the fluorescence plots, black and red curves represent positive and negative samples.

To optimize the additional pre-incubation step in our assay, we examined both the pre-incubation time and the crRNA configuration for contaminant removal. This optimization was designed to achieve efficient Cas12a-mediated *cis*-cleavage of contaminating amplicons, converting the intact contaminant into smaller fragments that would no longer function as templates for subsequent amplification, while minimizing undesired *trans*-cleavage of primers. As shown in Figure 2a, efficient cleavage of the intact contaminant amplicon was achieved within 10 min, whereas longer incubation did not yield an obvious additional benefit. Notably, previous studies showed that the persistence of Cas12a *trans* activity resulted in stoichiometric cleavage products accruing with longer working times. However, a short reaction time reduced any unwanted *trans* activity [22,23]. Several researchers have proposed that multiplex CRISPR/crRNA strategies can confer high levels of sensitivity and specificity [24,25,26]. To further improve contaminant removal, we compared single-crRNA and dual-crRNA systems. The dual-crRNA design, targeting two sites within the same contaminant fragment, showed stronger cleavage efficiency than either single-crRNA condition (Figure 2a and Figure S4). Furthermore, the introduced multi-Cas12a-crRNA system was demonstrated to specifically degrade contaminants without affecting the target template (Figure S5).

Similarly, because the on-target and *trans*-cleavage mechanisms of Cas12a are also dependent on Mg^2+^, we investigated the concentration of Mg^2+^ in the reaction [23]. Reducing the Mg^2+^ concentration significantly attenuated non-target ssDNA (primer) degradation (Figure 2b). At 1.5 mM Mg^2+^, little primer degradation was observed in the PCR. Although target cleavage may have been slower when using lower Mg^2+^ concentrations, all target-contaminant cleavage was still achieved at 1.5 mM Mg^2+^ after 10 min (Figure S6). This suggested that a concentration of 1.5 mM Mg^2+^ may help minimize *trans*-cleavage activity of Cas12a while maintaining its *cis*-cleavage activity. Subsequently, we also compared the dependence of CRISPR/Cas12a cleavage on buffers 1 and 2, which were Cas12a and PCR buffers, respectively. As shown in Figure S7, the cleavage efficiency of Cas12a was comparable under both buffering conditions. In addition, we evaluated the ratio of crRNA to Cas12a according to the degradation efficiency of the contaminants. As shown in Figure S8, the optimal ratio of crRNA to Cas12a was 1:1.

Generally, a very small amount of residual contamination in the air in a routine work environment can lead to false-positive results. Therefore, a series of simulated contaminants from *L. monocytogenes* was used to investigate the capacity of Cas12a to overcome this cross-contamination. As shown in Figure 2c, in the system without Cas12a, we observed amplification as low as 10^1^ copies of the contaminant. In contrast, the system with Cas12a prevented the amplification of up to 10^7^ copies of carryover contaminant DNA (Figure 2c, lower panel). These results clearly demonstrated that the Cas12a pre-incubation step effectively eliminated PCR contaminants under experimental conditions. This finding is particularly important, as these results demonstrated that even minuscule amounts of contaminant nucleic acids can produce unwanted amplification. We also showed that the Cas12a-crRNA system reduced false-positive results caused by contamination. This was confirmed by performing Cas12a-free PCR reactions and Cas12a-treated PCR reactions in 10 copies of target DNA and simulating a range of carryover contaminant amplicons in the samples that mimicked a type of field contamination (e.g., a situation in which target DNA is scarce and background contamination is present). The Cas12a-free reactions amplified the nontargeted negative control DNA, generating false-positive amplification signals indistinguishable from real DNA signals. In contrast, PAM-PCR eliminated these false positives, and we were able to clearly distinguish the target (10 copies) from samples carrying contaminants of up to 10^7^ copies/μL (Figure S9). These results demonstrate the robustness of the CRISPR/Cas12a system, which efficiently removed DNA contaminants even when the target DNA was at very low concentrations, ensuring accurate results.

Based on these results, we further investigated the practical application of Cas12a for degrading contaminants containing PAM sites. We observed that negative control samples, in the absence of PCR cross-contamination, did not produce significant false-positive amplifications. However, in the presence of PCR cross-contamination, the negative control samples exhibited a significant amplification curve, leading to false-positive results. In contrast, when an additional pre-incubation step was performed before the PCR, the false positive results in the system disappeared, indicating that the contamination-cleavage ability of Cas12a was effective and that positive amplification was able to proceed without any adverse effects (Figure 2d).

### 3.3. Establishment of a Visual Single-Tube PCR Assay

**Figure 3:**
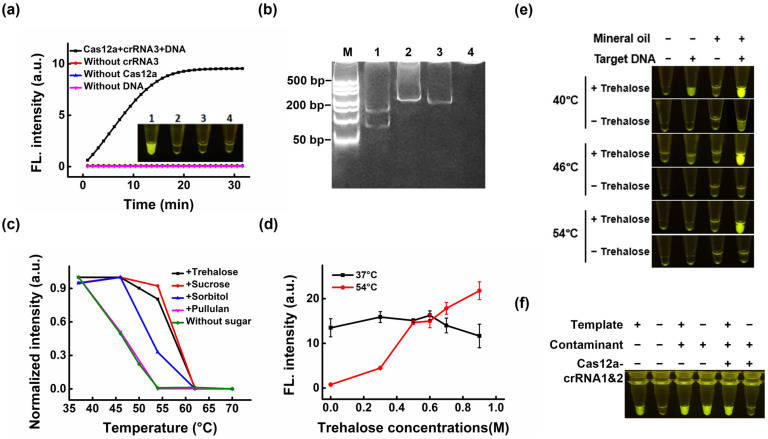
Establishment of a visual single-tube PCR assay. (**a**) Fluorescence analysis and (**b**) gel analysis of target PCR amplicon-activated Cas12a reactions. 1–4 indicate reactions without Cas12a, without crRNA-3, or without DNA, respectively. (**c**) Effects of four sugars on the thermal stability of Cas12a at different reaction temperatures. The control reaction was performed without sugar. (**d**) Effects of trehalose concentrations on the thermal stability of the Cas12a enzyme at 37 °C and 54 °C. (**e**) Endpoint fluorescence images of the one-pot PCR-Cas12a assay under different instrument temperature settings. Reactions were performed with the PCR instrument cap temperature set to 40 °C, 46 °C, or 54 °C, in the presence or absence of trehalose, mineral oil, and target DNA, as indicated. (**f**) Evaluation of the one-pot Cas12a-based platform for contamination-free visual detection. Tubes 1–2 show conventional PCR without contamination. Tubes 3–4 show contaminated reactions containing residual amplicons, in which the negative control produces a false-positive signal. Tubes 5–6 show that Cas12a-mediated decontamination eliminates the false-positive signal while preserving correct detection of the true template. “+” and “−” indicate the presence and absence of the indicated component, respectively.

To minimize carryover contamination that may occur when opening the tube, a Cas12a-detection complex (comprising Cas12a, crRNA3, trehalose, and the fluorophore–quencher-labeled ssDNA probe) present in the tube cover was used to detect the amplification products. As shown in Figure 3a, only the reaction mixture that contained the target DNA, Cas12a, and crRNA3 produced an ultra-bright fluorescent signal that was directly visualized under blue LED light, indicating that fluorescence generation depended on target-triggered activation of the *trans*-cleavage activity of Cas12a, which enabled nonspecific cleavage of the ssDNA probe. In addition, pronounced levels of cleavage fragments were visible (Figure 3b).

Generally, the Cas12a protein, as a thermolabile enzyme that typically operates at 37 °C, cannot tolerate a high PCR temperature of 95 °C. Some researchers have demonstrated that certain carbohydrates can be used as excipients to protect protein molecules from thermal denaturation. For example, the addition of trehalose was shown to maintain the normal activity of enzymes at high temperatures and may have improved the activity of these enzymes [27,28,29,30]. Therefore, to test the influence of different sugar solutions on the thermal stability of the Cas12a protein at various reaction temperatures, the Cas12a-crRNA complex was incubated in NEBuffer 2.1 for 10 min at temperatures ranging from 37 °C to 70 °C, with trehalose, sucrose, sorbitol, or pullulan, or without a sugar solution (Figure 3c). When incubated at 50 °C, the addition of three sugars had a heat-stabilizing effect on Cas12a, with trehalose and sucrose exhibiting the best performance. However, within the test temperature range (37–70 °C), the fluorescence intensity of Cas12a with pullulan solution was similar to that without sugar addition, indicating that the addition of pullulan solution did not have any marked thermostabilizing effect. This may be because pullulan typically exerts its protective effects in solid states, such as tablet or film forms, to highly restrict the molecular movement of proteins [31,32,33,34]. The thermal stability of this enzyme varied considerably with the addition of sugar solution at 54 °C. Although some sugars effectively improved the thermal stability of Cas12a, all activity was lost after reacting at 54 °C for more than 1 h (Figure S10). Despite this limitation, the addition of these sugar solutions reduced the rate of enzyme thermal inactivation to some extent. To further investigate the thermal protection of the Cas enzyme by trehalose solution, we also optimized the concentration of trehalose solution, as a concentration that was too low would result in insufficient hydrogen bond substitutions (due to water removal), while a concentration that was too high would result in insufficient hydrogen bonding on the protein surface (due to crystallization) [35]. At 37 °C, with increasing trehalose concentrations, the *trans*-cleavage fluorescent signal tended to be stable. This result confirmed that the addition of trehalose to the assay system did not interfere with the fluorescence signal or cleavage efficiency, ensuring that the enhanced signals observed at higher temperatures were solely due to improved enzyme stability. At 54 °C, with increasing trehalose concentrations, the fluorescent signal was gradually enhanced (Figure 3d). This was consistent with prior reports that trehalose stabilizes enzymes at high temperatures, reducing thermal inactivation [27]. Meanwhile, at a trehalose concentration of 0.6 M, the fluorescent signal was close to that at 37 °C, which was comparable to the maximum concentration documented for yeast cells in the literature [36].

### 3.4. Closed-Tube Visual Detection of Contamination-Free PCR Amplification

To further mitigate contamination risk, we created a one-pot approach that integrated template amplification with the Cas12a-detection complex reaction. In our evaluation of the PCR-Cas12a-based single-tube assay, we found that trehalose alone was insufficient to preserve Cas enzyme activity, as no fluorescence was visible after amplification. To overcome the problem of temperature incompatibility, we took advantage of the ability of trehalose solution to improve the thermal stability of Cas12a, combined with mineral oil insulation and temperature control of the cap of the PCR instrument, thus preventing the inactivation of the Cas12a protein located within the tube cap. We found that the PCR-Cas12a-based one-pot method was able to produce a bright yellow-green fluorescent signal (Figure 3e). Similarly, we applied this one-pot method to the decontamination and visualization tests of the Cas12a system, confirming that the combination of cross-contamination removal, PCR amplification, and fluorescence visualization in the closed tube was successful (Figure 3f). As shown in Figure 3f, the high fluorescence intensity observed in both Tube 3 (positive) and Tube 4 (negative control under contaminated conditions) highlights the impact of carryover contamination, where residual amplicons act as false-positive. However, after Cas12a-mediated pre-incubation, the false-positive signal (Tube 6) was successfully eliminated, while the true target (Tube 5) remained detectable. This confirms that the fluorescence is strictly template-dependent and that our platform effectively distinguishes true targets from contamination.

The sensitivity of the Cas12a-PCR system was further tested by performing a 10-fold gradient dilution of *L. monocytogenes*. Compared with the traditional PCR method, this method was able to successfully detect down to 100 copies of *L. monocytogenes* samples (Figure S11). Importantly, the Cas12a-treated PCR method could identify genomic samples without producing off-target signals, demonstrating its high specificity even in the presence of other pathogenic bacteria, such as *Staphylococcus aureus* (*S. aureus*), *Salmonella* spp., *Escherichia coli*, and *Enterobacter sakazakii* (*E. sakazakii*). As a proof-of-concept, we further evaluated the contaminant cleavage ability of the Cas12a-crRNA assay in actual sample analysis by measuring *L. monocytogenes* spiked in an artificially contaminated milk sample. The results showed that our proposed method achieved comparable results to the UDG method, which is a widely used decontamination strategy in practice (Figure S12). Therefore, this method has demonstrated its potential for use with complex samples.

### 3.5. Application of Cas12a-crRNA for Digesting Carryover Contamination in a LAMP Assay

**Figure 4:**
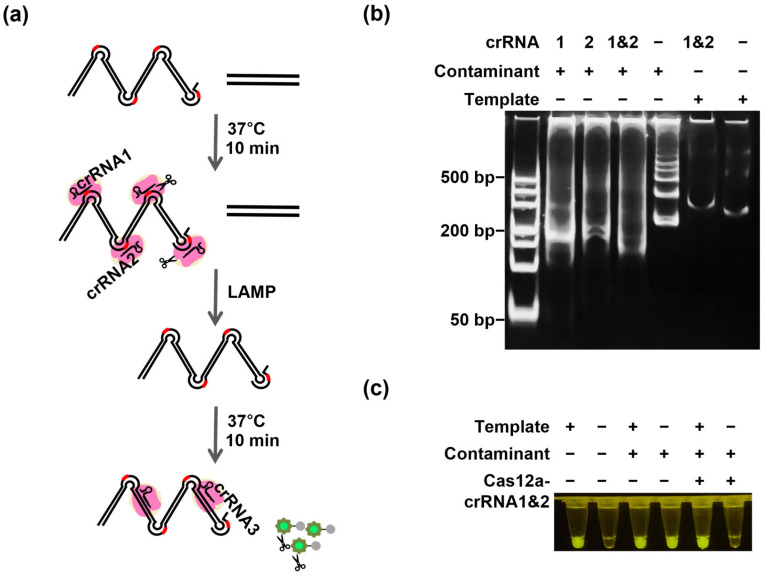
Application of Cas12a-crRNA to digest carryover contamination in a LAMP amplification. (**a**) Schematic overview of the CRISPR/Cas-LAMP assay. (**b**) Gel analysis of Cas12a-mediated contaminant cleavage in the LAMP system. Contaminant amplicons containing the engineered PAM site were treated with Cas12a-crRNA1, Cas12a-crRNA2, dual Cas12a-crRNAs (1 & 2), or no crRNA, while the authentic target template lacking the corresponding PAM site was analyzed in parallel. (**c**) Closed-tube visual detection of contamination-free LAMP amplification. Tubes 1–2 show conventional PCR without contamination. Tubes 3–4 show contaminated reactions containing residual amplicons, in which the negative control produces a false-positive signal. Tubes 5–6 show that Cas12a-mediated decontamination eliminates the false-positive signal while preserving correct detection of the true template. “+” and “−” indicate the presence and absence of the indicated component, respectively.

Similarly, Cas12a-based methods can also be used in LAMP detection to eliminate cross-contamination (Figure 4a). LAMP, as a convenient constant-temperature method suitable for field testing, requires test templates to be placed in closed tubes, thereby preventing cross-contamination. Because the inner primer in the LAMP reaction contains a TTT spacer, it can bind specifically to Cas12a without any special primer engineering design or modification, adding convenience without affecting specificity or amplification [37]. Because the Mg^2+^ concentration affected both the cleavage efficiency and the LAMP amplification efficiency, a CRISPR/Cas-LAMP assay was performed with 6 μM Mg^2+^ (Figure S13). Significant product degradation was only observed in the presence of contaminants (Figure 4b). Good degradation of residual contaminants stemming from prior amplification was achieved, which effectively avoided target amplification of up to 10^6^ copies of contaminant DNA (Figures S14 and S15). Moreover, the effects of Cas12a-LAMP assay specificity and sensitivity on the accuracy of the results were evaluated. The real-time fluorescence kinetic curve demonstrated that this method could detect as few as 100 copies/μL, and no non-targeted signals appeared even when other pathogenic bacteria were present, proving its robust specificity (Figure S16). Importantly, the target was easily visually identified when the Cas12a-crRNA3 complex was pre-positioned in the tube cap (Figure 4c). These results clearly show that CRISPR/Cas12a can be used to eliminate contamination in LAMP assays and demonstrate the potential of one-pot detection methods.

### 3.6. Discussion

We have developed a novel one-pot decontamination strategy for PCR and LAMP, utilizing the dual activities of Cas12a. During the initial stage, the system maximizes cis-cleavage to degrade contaminants while minimizing trans-cleavage to prevent nonspecific degradation of single-stranded primers. After amplification, the trans-cleavage activity of the Cas12a-crRNA3 complex, released from the tube cap, is activated to enable sensitive visual detection. This strategy ensures both effective contamination removal and accurate detection. Compared to other methods that combine contamination removal with endpoint detection, our method has significant advantages in the types of contaminant amplicons, detection time, and ability to remove contaminants. Furthermore, it only requires a single Cas12a protein to perform two functions in one pot, namely contaminant removal and visual detection (Table S4) [9,13,14,18,19,20,38,39,40,41]. When the contaminant sequence is known, we can design specific crRNAs with appropriate PAM sites based on its specific DNA. However, a potential limitation of this approach is the reliance on sequencing for unknown sources of contamination before designing corresponding crRNAs and primers [42]. In short, our research has proposed a reliable decontamination strategy, demonstrating that a single Cas12a enzyme is sufficient to achieve contaminant removal and visual detection in a single tube.

## 4. Conclusions

In conclusion, we have demonstrated a decontamination strategy that enabled contaminant removal and visual detection in a single-tube reaction by combining Cas12a with PCR/LAMP. This approach is very intuitive, fast, and convenient, requiring only the addition of a pre-incubation step before amplification. An elimination rate of 10^6^ copies was achieved by degrading the contaminant amplicon, with an overall detection time of under 1 h. It is worth noting that the method takes advantage of the thermoprotective effect of trehalose on Cas enzymes, allowing a reaction temperature of 54 °C. In addition, our one-pot, one-step strategy used trehalose to improve the thermal stability of the Cas12a protein, along with mineral oil-based insulation and tube cap temperature regulation, preventing the PCR instrument from inactivating the Cas12a protein in the tube cap. This triple protection eliminated cross-contamination, facilitated PCR/LAMP amplification, and generated a visible fluorescent signal in a closed tube. Future directions include integrating this system with lateral flow assay (LFA) strips for direct visual detection without the need for external LED lights.

## Data Availability

The original contributions presented in this study are included in the article/Supplementary Materials. Further inquiries can be directed to the corresponding author.

## Supplementary Materials

The following supporting information can be downloaded at: https://www.mdpi.com/article/10.3390/bios16030170/s1, Figure S1: Schematic diagram of Cas12a- and Cas9-based decontamination strategy. (a,b) PCR decontamination strategies. For Cas12a (a), efficient elimination is achieved regardless of PAM position (5′ or 3′ end) because its cleavage site is 18 nt away from the PAM, leaving residual fragments too short to stably re-anneal with primers. In contrast, Cas9 (b) cleaves only 3 nt from the PAM; when the PAM is at the 5′ end (I), the remaining 17 nt fragment poses a re-extension and contamination risk. (c) LAMP decontamination strategies. Both Cas12a (c) and Cas9 (d) digest amplicons, but the short cleavage gap of Cas9 (~3 nt) may leave fragments capable of triggering false-positive amplification; Figure S2: Specific locations of primers and crRNAs designed for *L. monocytogenes* targets. The red sequences indicate the PAM sites, and the blue sequences represent the target sequences of crRNA (crRNA 1, crRNA 2, and crRNA 3); Figure S3: The feasibility of the PAM-PCR assay. Comparison of PAM- primers and normal primers in PCR amplifications (a), with a gel image (b); Figure S4: Schematic illustration and evaluation of contaminant cleavage by Cas12a with different crRNA designs. (a) Schematic diagram of Cas12a-mediated cleavage of the contaminant amplicon directed by crRNA1 and crRNA2. Cleavage with either crRNA generates 26-bp and 150-bp fragments, whereas simultaneous use of both crRNAs additionally produces a 124-bp fragment. (b) Real-time amplification analysis of contaminant degradation by Cas12a in the presence of crRNA1, crRNA2, or dual crRNAs (1&2); Figure S5: Gel analysis of specific degradation of contaminant DNA by Cas12a-crRNA1&2 while preserving the target template; Figure S6: Gel analysis of contaminant degradation by Cas12a at different Mg^2+^ concentrations. Reactions were performed with Cas12a-crRNA at the indicated Mg^2+^ concentrations, and a reaction without Cas12a-crRNA was included as a control; Figure S7: Gel analysis of Cas12a-mediated contaminant degradation in different buffers. Buffer 1 and Buffer 2 denote the Cas12a reaction buffer and PCR buffer, respectively; Figure S8: Gel analysis of contaminant degradation by Cas12a at different Cas12: dual-crRNA ratios. In the dual-crRNA system, each crRNA was used at 0.05 μM; Figure S9: Real-time amplification analysis of carryover contaminants in Cas12a-free and Cas12a-treated PCR reactions containing 10 copies of target DNA. Cas12a-free PCR (a) and Cas12a-treated PCR (b) were evaluated using a simulated series of contaminants (10^7^ to 0 copies/μL). NTC denotes the no-template control; Figure S10: Effect of different sugars on the thermal stability of Cas12a at 54 °C. Normalized fluorescence of the Cas12a-crRNA complex was recorded over time in the presence of trehalose, sucrose, sorbitol, or without sugar; Figure S11: Sensitivity and specificity of Cas12a-PCR for detection of *L. monocytogenes*. (a,b) Real-time amplification curves of 10-fold serial dilutions of *L. monocytogenes* in Cas12a-free PCR (a) and Cas12a-treated PCR (b). (c,d) Real-time amplification curves showing the specificity of Cas12a-free PCR (c) and Cas12a-treated PCR (d) in the presence of *L. monocytogenes*, *S. aureus*, *Salmonella* spp., *Escherichia coli*, and *E. sakazakii*. NTC denotes the no-template control; Figure S12: Evaluation of different decontamination methods in an artificially *L. monocytogenes*-contaminated milk sample. (a) Real-time amplification analysis of positive and negative milk samples with or without Cas12a treatment. (b) Real-time amplification analysis of positive and negative milk samples with or without UDG treatment; Figure S13: Effect of Cas12a *trans*-cleavage on LAMP reaction primers at various Mg^2+^ concentrations in the presence of a contaminant; Figure S14: Evaluation of the practical ability of adding Cas12a to eliminate LAMP false-positive results due to carryover contamination. Conventional LAMP results without cross-contamination (left panel). False-positive results due to contamination from previous reactions as a template (middle panel). Results of eliminating false-positive amplification by adding Cas12a to cleave contaminants containing PAM sites (right panel); Figure S15: Test results of the system cleavage capacity in the presence and absence of Cas12a at different contaminant concentrations. (a) Cas12a-free reactions exhibited amplification in the presence of as few as 10^1^ copies of the carryover contaminant, and (b) Cas12a treatment effectively eliminated the amplification; Figure S16: Sensitivity and specificity of Cas12a-LAMP for detection of *L. monocytogenes*. (a,b) Real-time amplification curves of 10-fold serial dilutions of *L. monocytogenes* in Cas12a-free LAMP (a) and Cas12a-treated LAMP (b). (c,d) Real-time amplification curves showing the specificity of Cas12a-free LAMP (c) and Cas12a-treated LAMP (d) in the presence of *L. monocytogenes*, *S. aureus*, *Salmonella* spp., *Escherichia coli*, and *E. sakazakii*. NTC denotes the no-template control.; Table S1: Sequences of PCR primer; Table S2: Sequences of LAMP primer; Table S3: crRNA and probe sequences; Table S4: Target gene sequences; Table S5: Comparison of different methods for contamination removal and endpoint detection.
