# Supplementary material for: A One-Pot CRISPR/Cas12a-Based Platform for Contamination-Free Nucleic Acid Amplification Detection
Source: Biosensors (Basel). 2026 Mar 19;16(3):170. doi: 10.3390/bios16030170 (PMC13023935; doi:10.3390/bios16030170)
Supplement: Supplementary file 1 [file biosensors-16-00170-s001.zip › biosensors-4164725-supplementary.pdf]

Supporting Information

A one-pot CRISPR/Cas12a-based platform for contamination-free nucleic acid amplification detection

Wei Tan-tai, Qin-feng Xu,* Wen-juan Zhang, Yan-ni Li, Hao Liu
School of Food Science and Engineering, National R&D Center for Goat Dairy Products Processing Technology, Shaanxi University of Science and Technology, Xi'an, Shaanxi 710021, China
*Corresponding author: Qin-feng Xu
Addresses: School of Food Science and Engineering, National R&D Center for Goat Dairy Products Processing Technology, Shaanxi University of Science and Technology, Xi'an, Shaanxi 710021, China
Tel.: +86 18821682062
E-mail addresses: xuqinfeng@sust.edu.cn.

Table S1. Sequences of PCR Primer

Pathogen	Primer sequence (5′–3′) [a]		Size (nt)
Listeria monocytogenes (*L. monocytogenes*)	FP	GTCTTTTAAGTGGAGTAAACCTT	23
	RP	ACAAGACTTCACCAATCCA	19
	PAM-FP	TGGATTTCAGGCAAGTCATCTTGTTCG	27
	PAM-RP	TCCATTTCACGTGTACACAGAAAAGCG	27

[a] FP, forward primer; RP, reverse primer.

Table S2. Sequences of LAMP primer

Pathogen	Primer sequence (5′–3′) [a]		Size (nt)
Listeria monocytogenes (*L. monocytogenes*)	F3	GTCTTTTAAGTGGAGTAAACCTT	23
	B3	ACAAGACTTCACCAATCCA	19
	FIP	CCTGTGCCAAAGCATTTTTACATTTTTTA GGCAAGTCATCTTGTTCG	47
	BIP	TAAGTCTCTTTGCAATTGACCGACTTTTA CGTGTACACAGAAAAGCG	47

[a] F3, forward primer; B3, backward primer; FIP, forward inner primer; BIP, backward inner primer.

Table S3. crRNA and probe sequences

Name[a]	Complete crRNA sequence	Size (nt)
crRNA1	UAAUUUCUACUAAGUGUAGAUAGGCAAGUCAUCU UGUUCGAUUAA	45
crRNA2	UAAUUUCUACUAAGUGUAGAUACGUGUACACAGA AAAGCGCUGAU	45
crRNA3	UAAUUUCUACUAAGUGUAGAUAGUCUCUUUGCAA UUGACCGACGU	45
Probe	FAM-TTATT-BHQ	5

[a] crRNA1 and crRNA2 used for eliminating contamination; crRNA3 used for detection.

Table S4. Target gene sequences

Pathogen	Target sequence (5′–3′)	Size (bp)
Listeria monocytogenes (*L. monocytogenes*)	GTCTTTTAAGTGGAGTAAACCTTTTTGAACGTGGATAGGCA AGTCATCTTGTTCGATTAATATATAATTAGCCGTTTTGGTTT TATAATGTAAAAATGCTTTGGCACAGGCTAGTTTTAAGTCT CTTTGCAATTGACCGACGTTCGCACTTGCATGATAAAGTAA AAAAGCAATCAGCGCTTTTCTGTGTACACGTATGGATTGGT GAAGTCTTGT[a]	216
	TGGATTTCAGGCAAGTCATCTTGTTCGATTAATATATAATT AGCCGTTTTGGTTTTATAATGTAAAAATGCTTTGGCACAGG CTAGTTTTAAGTCTCTTTGCAATTGACCGACGTTCGCACTTG CATGATAAAGTAAAAAAGCAATCAGCGCTTTTCTGTGTAC ACGTGAAATGGA[b]	176
	GTCTTTTAAGTGGAGTAAACCTTTTTGAACGTGGATTTTAG GCAAGTCATCTTGTTCGATTAATATATAATTAGCCGTTTTGG TTTTATAATGTAAAAATGCTTTGGCACAGGCTAGTTTTAAG TCTCTTTGCAATTGACCGACGTTCGCACTTGCATGATAAAG TAAAAAAGCAATCAGCGCTTTTCTGTGTACACGTAAAATG GATTGGTGAAGTCTTGT[c]	222

[a] The PCR amplification sequence was amplified with FP and RP primers. This sequence then served as the template DNA.

[b] The contaminant DNA sequence was amplified using the PAM-FP and PAM-RP primers in the PCR.

[c] The LAMP reaction amplification sequence, which was the contaminant DNA sequence in the LAMP.

[b c] The TTT and AAA sequences in the red sections of the amplified sequence were introduced using primers that were designed to meet Cas12a's requirements, which necessitate the presence of a PAM site within the amplified sequence.

Table S5. Comparison of different methods for contamination removal and endpoint detection

System name	Types of enzymes	Amplification strategy	Signal output	Assay time	Capacity to remove contamination	One-pot assay	Reference
CUT-LAMP	Cas9; Cas12a	LAMP	Fluorescence / naked-eye	60 min	10 pg	No	[1]
CRISPR/Cas9 Eraser for PCR	Cas9	PCR/RT-PCR	Lateral flow device	77-113min	-	No	[2]
UDG-LAMP	UDG	LAMP	Fluorescence	65 min	100 ag	No	[3]
AUDG-SAMRS-LAMP	UDG	LAMP	Lateral flow	~95 min	10 fg	No	[4]
CADLAB	UDG; PfAgo	LAMP	Fluorescence	65 min	-	Yes	[5]
UCLD	UDG; Cas12a	LAMP	Fluorescence	105 min	-	No	[6]
UDG-LAMP-CRISPR/Cas12a	UDG; Cas12a	LAMP	Fluorescence	75 min	-	No	[7]
UDG-LAMP-CRISPR/Cas12b	UDG; Cas12b	LAMP	Fluorescence	30-55 min	1 pg	Yes	[8]
UPC-MRS	UDG; Cas12a	PCR	T2 signal	60 min	10-15 g	No	[9]
ETL-CRISPR	UDG; Cas12a	LAMP	Fluorescence/lateral flow	60 min	0.01 copies	No	[10]
-	Cas12a	PCR/LAMP	Fluorescence	60 min	106 copies (~1 pg)	Yes	This work

Note: -, not specified.

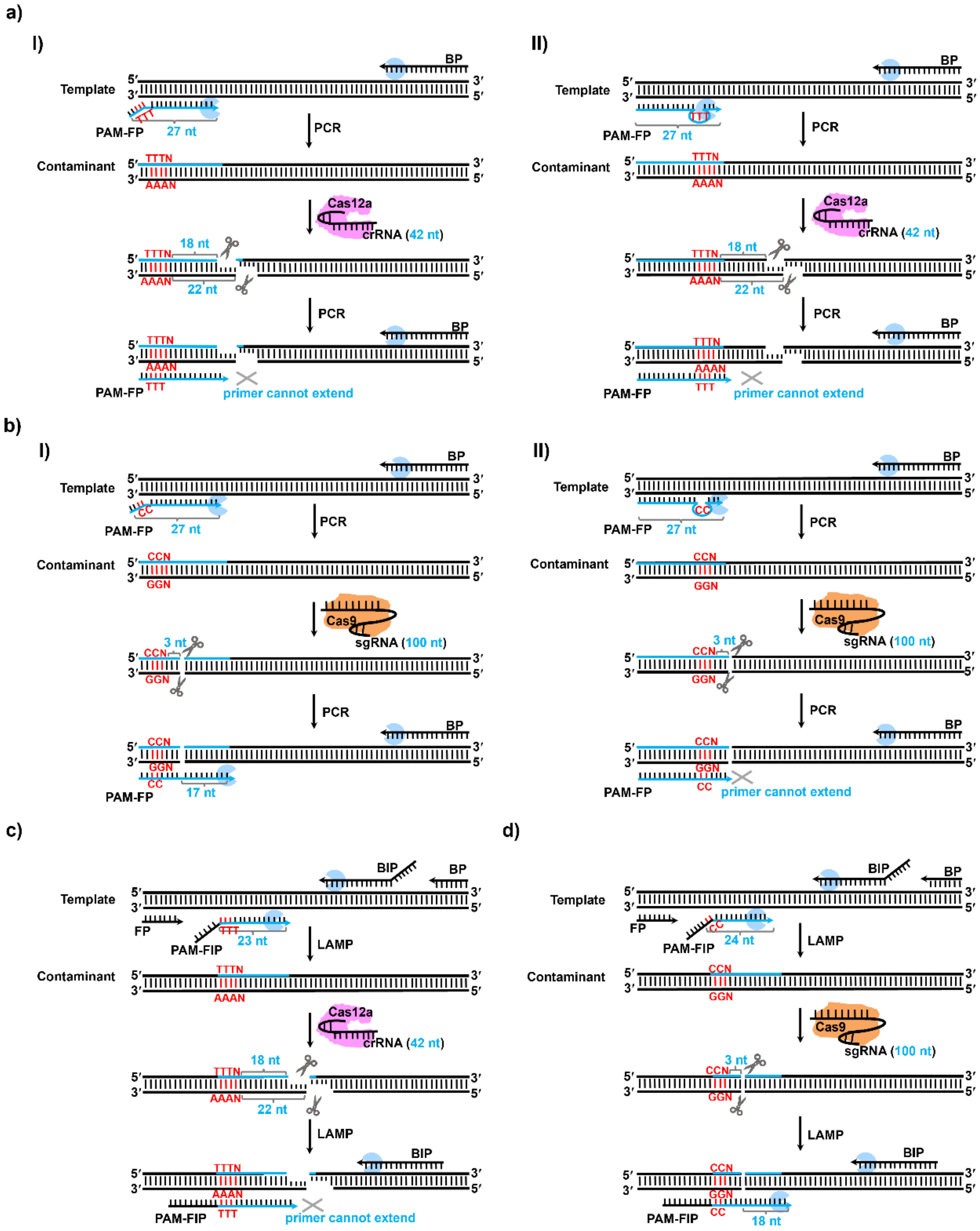

Figure S1. Schematic diagram of Cas12a- and Cas9-based decontamination strategy. (a-b) PCR decontamination strategies. For Cas12a (a), efficient elimination is achieved regardless of PAM position (5' or 3' end) because its cleavage site is 18 nt away from the PAM, leaving residual fragments too short to stably re-anneal with primers. In contrast, Cas9 (b) cleaves only 3 nt from the PAM; when the PAM is at the 5' end (I), the remaining 17 nt fragment poses a re-extension and contamination risk. (c) LAMP decontamination strategies. Both Cas12a (c) and Cas9 (d) digest amplicons, but the short cleavage gap of Cas9 (~3 nt) may leave fragments capable of triggering false-positive amplification.

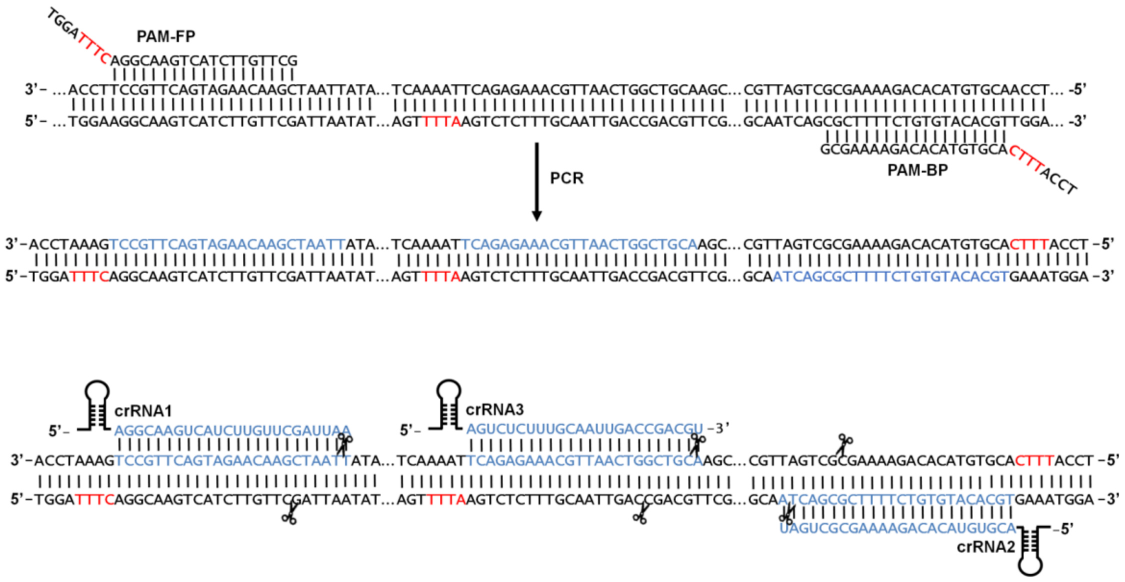

Figure S2. Specific locations of primers and crRNAs designed for *L. monocytogenes* targets. The red sequences indicate the PAM sites, and the blue sequences represent the target sequences of crRNA (crRNA 1, crRNA 2, and crRNA 3).

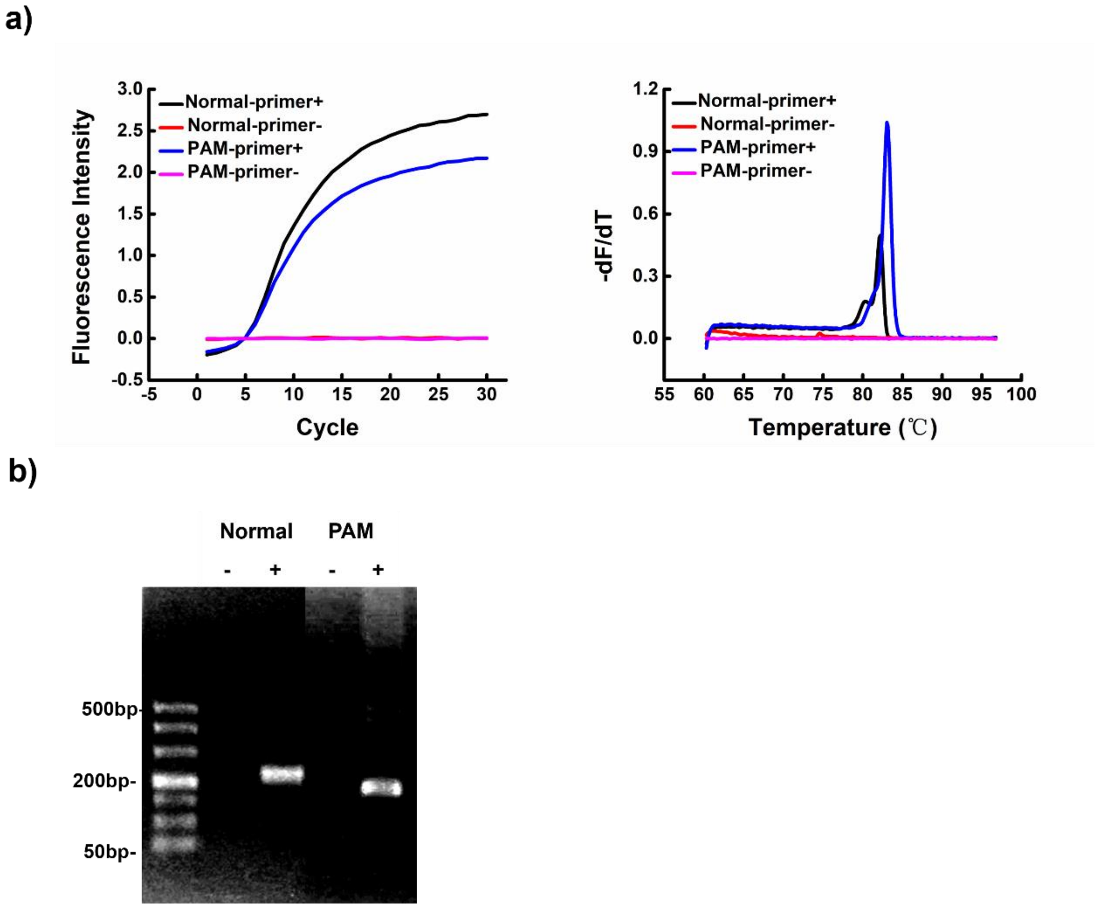

Figure S3. The feasibility of the PAM-PCR assay. Comparison of PAM- primers and normal primers in PCR amplifications (a), with a gel image (b).

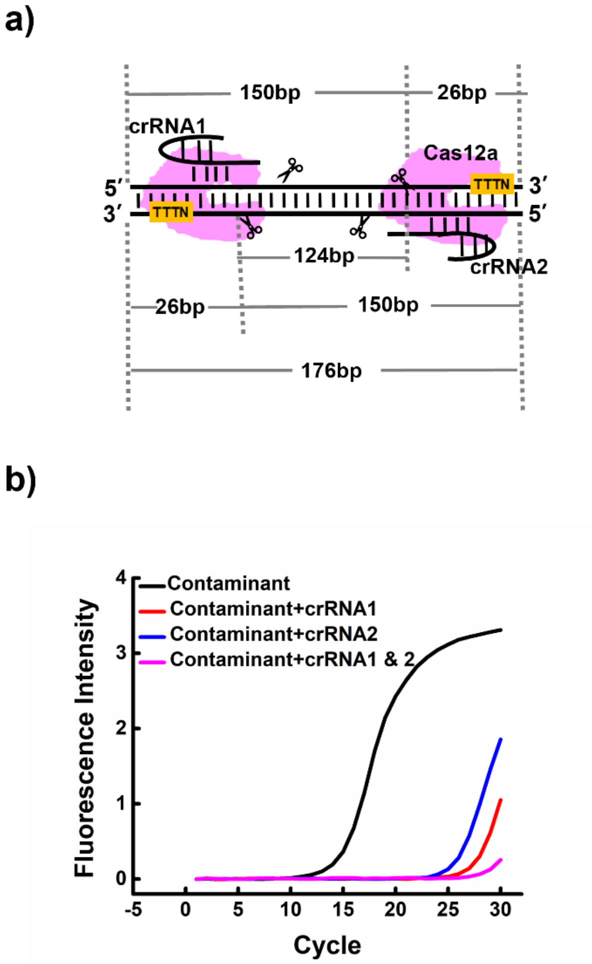

Figure S4. Schematic illustration and evaluation of contaminant cleavage by Cas12a with different crRNA designs. (a) Schematic diagram of Cas12a-mediated cleavage of the contaminant amplicon directed by crRNA1 and crRNA2. Cleavage with either crRNA generates 26-bp and 150-bp fragments, whereas simultaneous use of both crRNAs additionally produces a 124-bp fragment. (b) Real-time amplification analysis of contaminant degradation by Cas12a in the presence of crRNA1, crRNA2, or dual crRNAs (1&2).

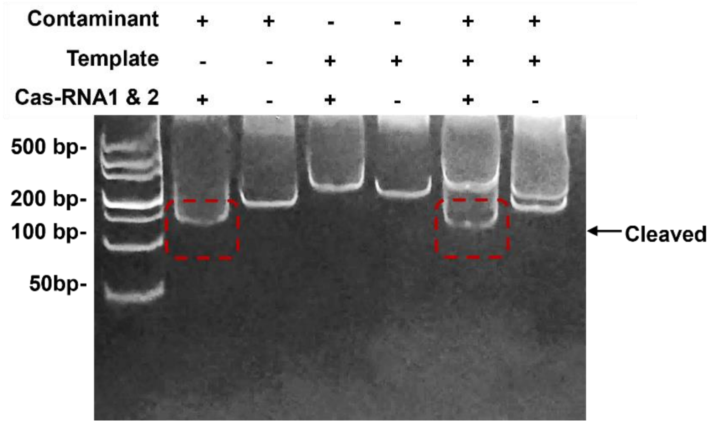

Figure S5. Gel analysis of specific degradation of contaminant DNA by Cas12a-crRNA1&2 while preserving the target template.

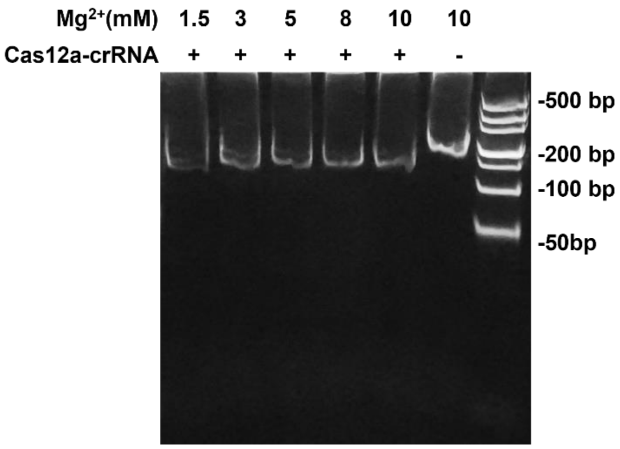

Figure S6. Gel analysis of contaminant degradation by Cas12a at different Mg^{2+} concentrations. Reactions were performed with Cas12a-crRNA at the indicated Mg^{2+} concentrations, and a reaction without Cas12a-crRNA was included as a control.

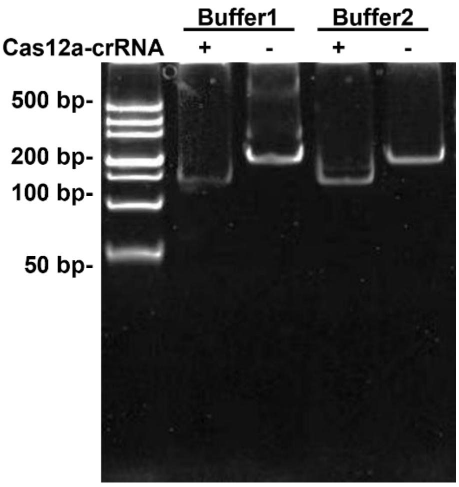

Figure S7. Gel analysis of Cas12a-mediated contaminant degradation in different buffers. Buffer 1 and Buffer 2 denote the Cas12a reaction buffer and PCR buffer, respectively.

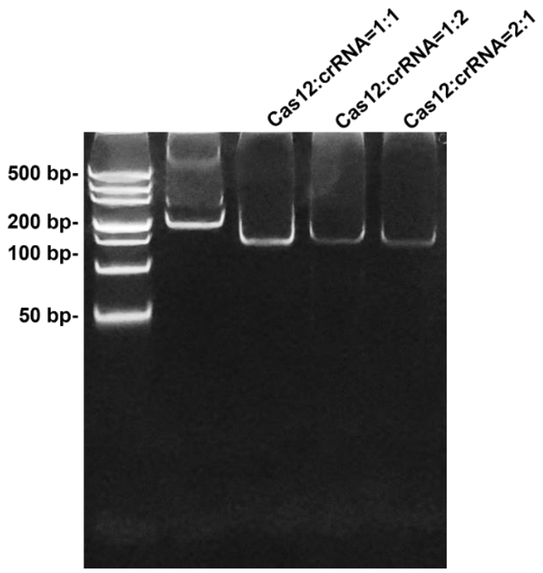

Figure S8. Gel analysis of contaminant degradation by Cas12a at different Cas12: dual-crRNA ratios. In the dual-crRNA system, each crRNA was used at 0.05 μM.

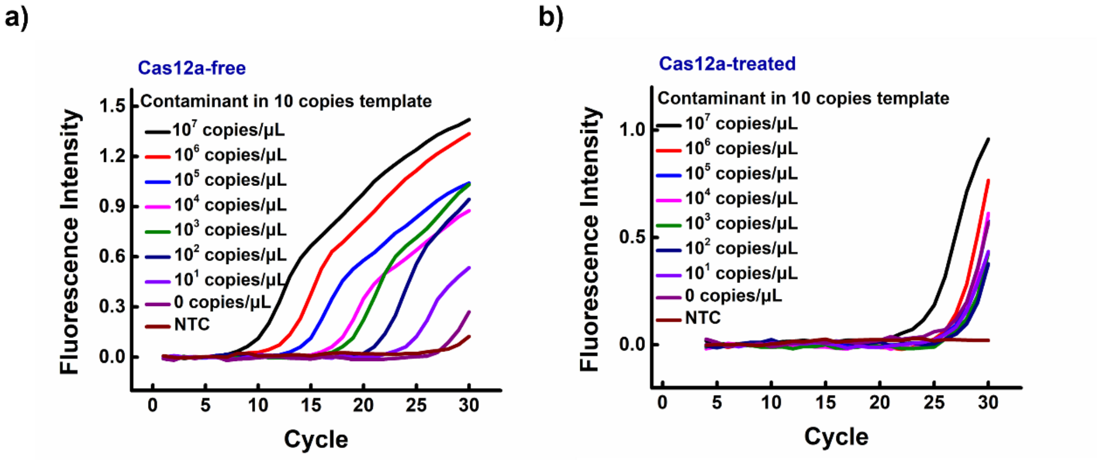

Figure S9. Real-time amplification analysis of carryover contaminants in Cas12a-free and Cas12a-treated PCR reactions containing 10 copies of target DNA. Cas12a-free PCR (a) and Cas12a-treated PCR (b) were evaluated using a simulated series of contaminants (10^7 to 0 copies/μL). NTC denotes the no-template control.

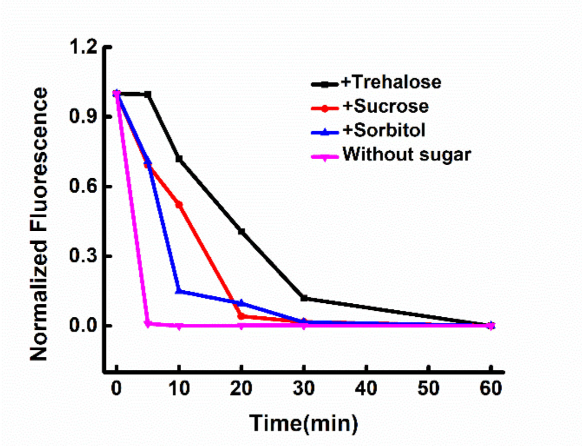

Figure S10. Effect of different sugars on the thermal stability of Cas12a at 54°C. Normalized fluorescence of the Cas12a-crRNA complex was recorded over time in the presence of trehalose, sucrose, sorbitol, or without sugar.

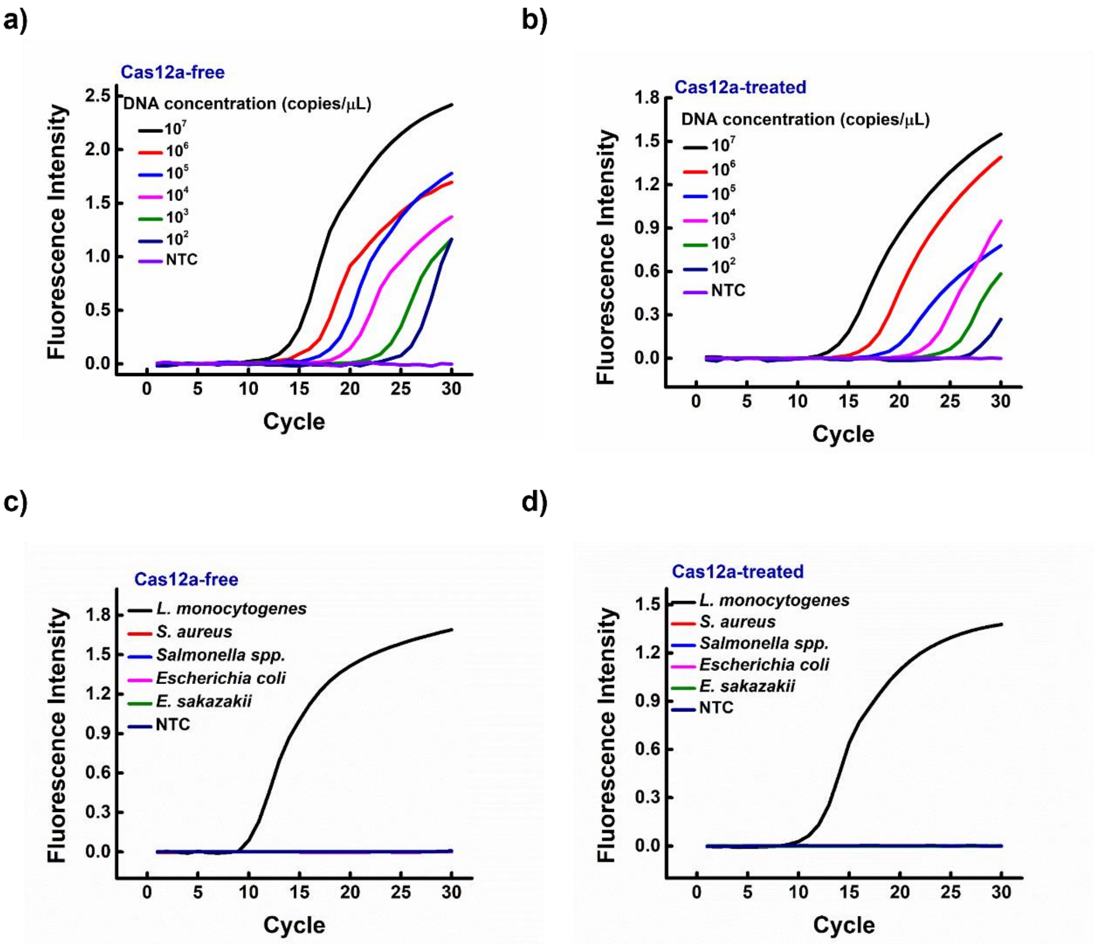

Figure S11. Sensitivity and specificity of Cas12a-PCR for detection of *L. monocytogenes*. (a,b) Real-time amplification curves of 10-fold serial dilutions of *L. monocytogenes* in Cas12a-free PCR (a) and Cas12a-treated PCR (b). (c,d) Real-time amplification curves showing the specificity of Cas12a-free PCR (c) and Cas12a-treated PCR (d) in the presence of *L. monocytogenes*, *S. aureus*, *Salmonella spp.*, *Escherichia coli*, and *E. sakazakii*. NTC denotes the no-template control.

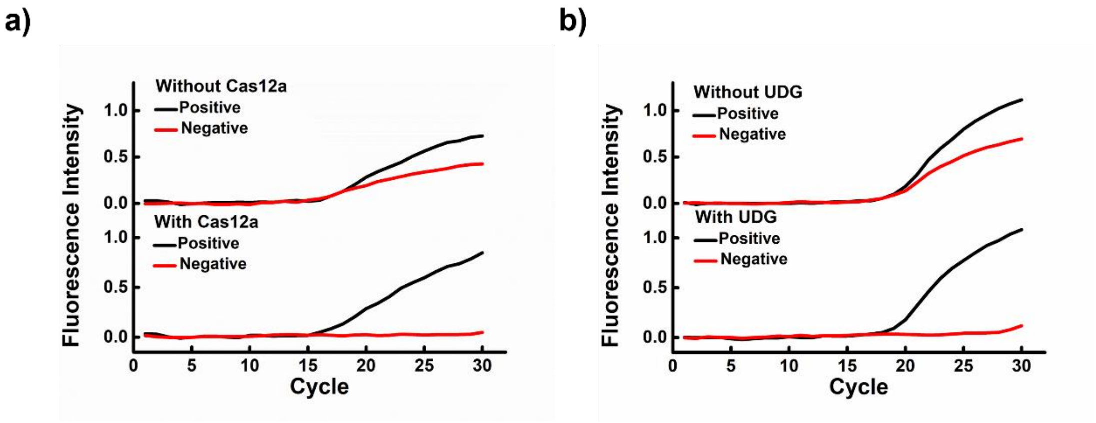

Figure S12. Comparison of Cas12a- and UDG-based decontamination strategies in artificially contaminated milk samples. (a) Real-time amplification analysis of positive and negative milk samples with or without Cas12a treatment. (b) Real-time amplification analysis of positive and negative milk samples with or without UDG treatment.

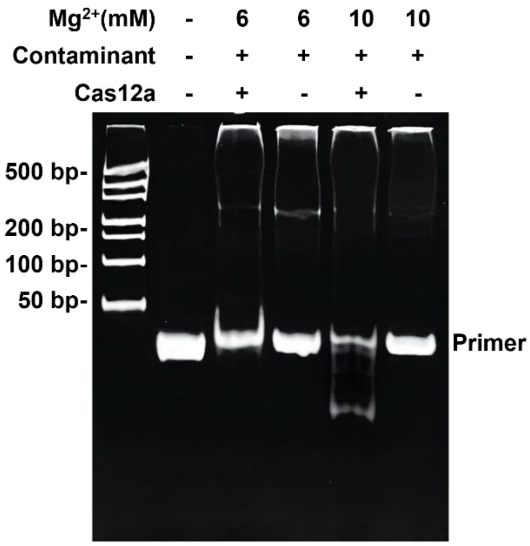

Figure S13. Effect of Cas12a *trans*-cleavage on LAMP reaction primers at various Mg^{2+} concentrations in the presence of a contaminant.

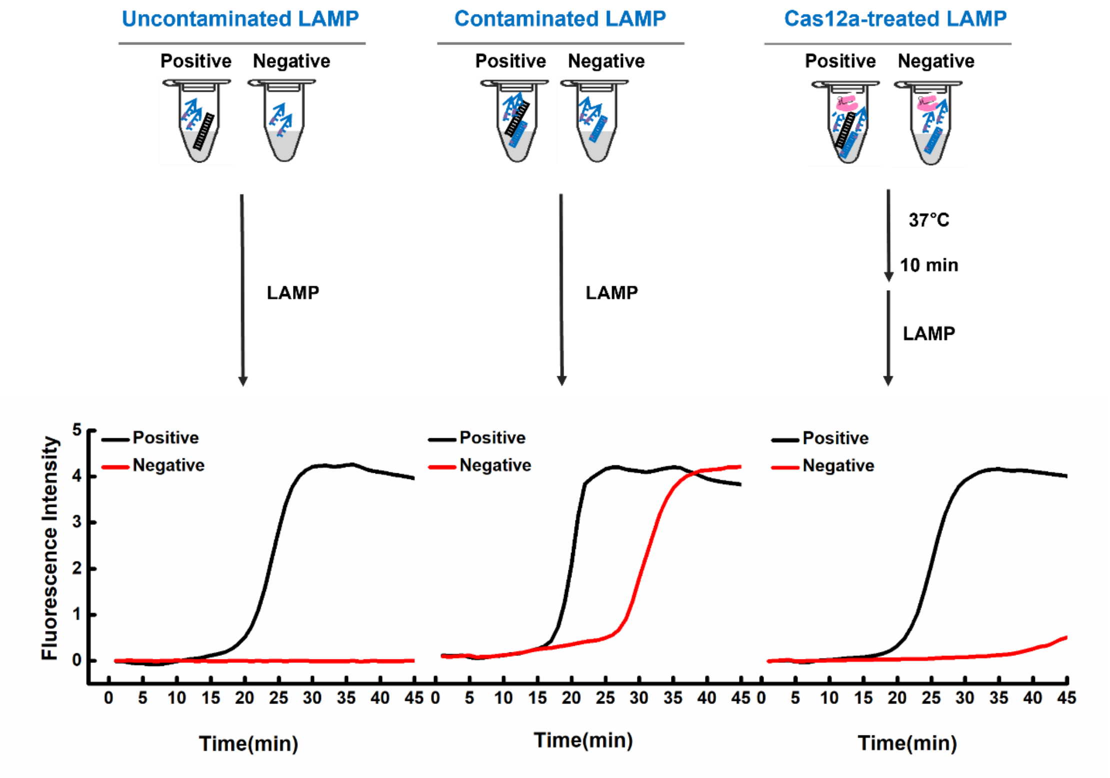

Figure S14. Evaluation of the practicality ability of adding Cas12a to eliminate LAMP false-positive results due to carryover contamination. Conventional LAMP results without cross-contamination (left panel). False-positive results due to contamination from previous reactions as a template (middle panel). Results of eliminating false-positive amplification by adding Cas12a to cleave contaminants containing PAM sites (right panel).

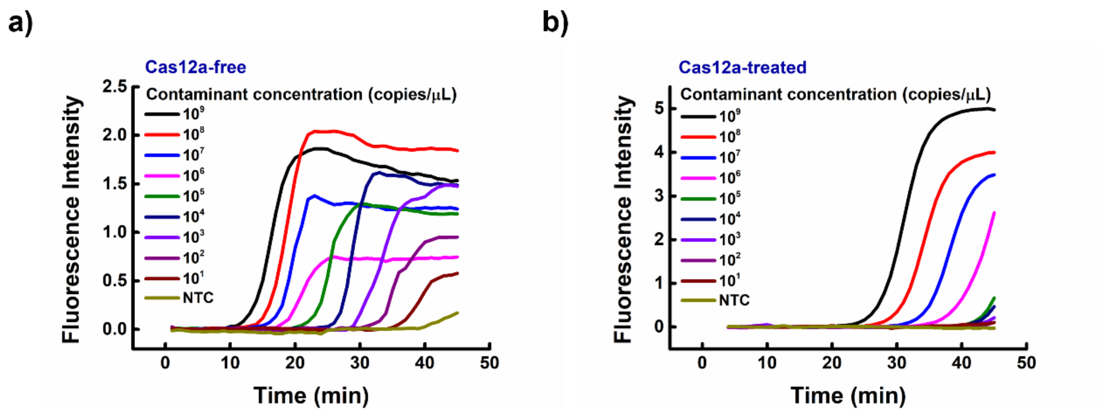

Figure S15. Test results of the system cleavage capacity in the presence and absence of Cas12a at different contaminant concentrations. (a) Cas12a-free reactions exhibited amplification in the presence of as few as 10^1 copies of the carryover contaminant, and (b) Cas12a treatment effectively eliminated the amplification.

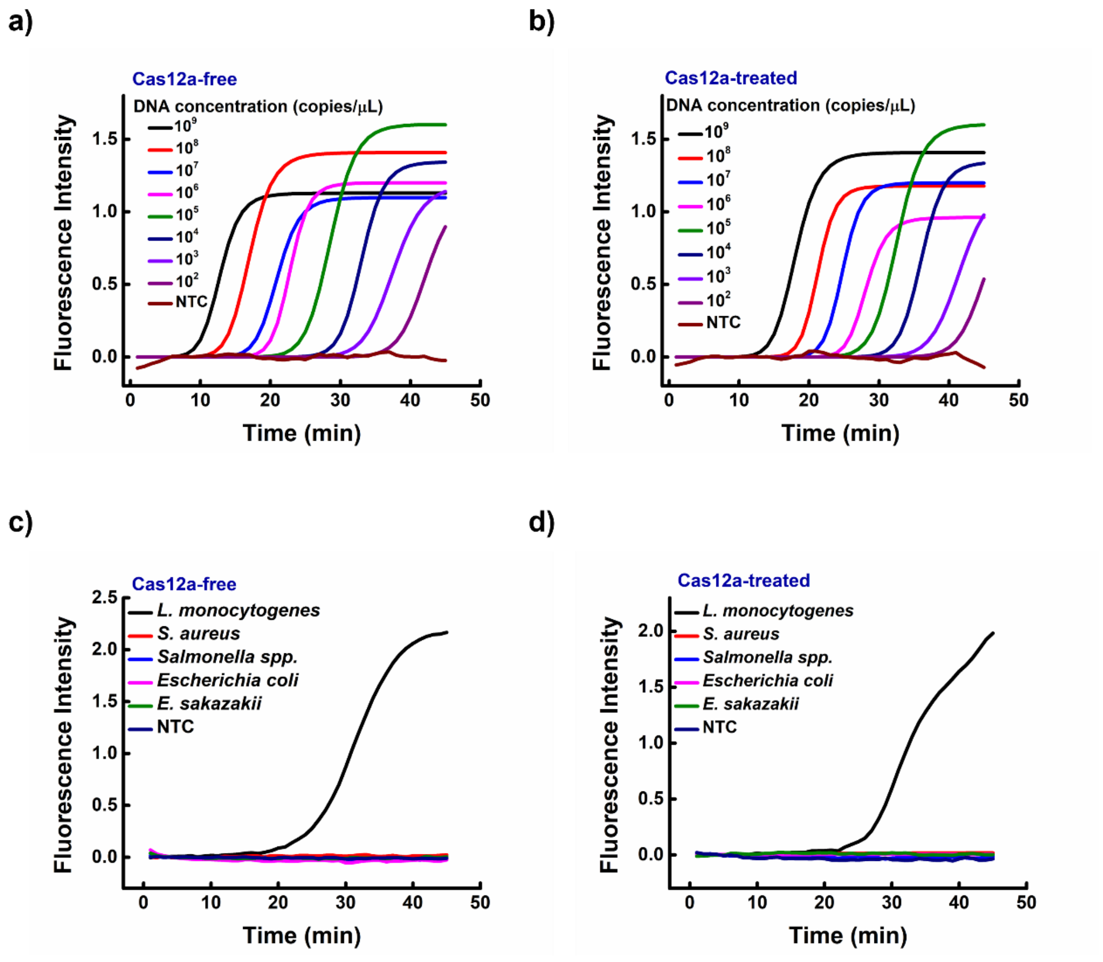

Figure S16. Sensitivity and specificity of Cas12a-LAMP for detection of *L. monocytogenes*. (a,b) Real-time amplification curves of 10-fold serial dilutions of *L. monocytogenes* in Cas12a-free LAMP (a) and Cas12a-treated LAMP (b). (c,d) Real-time amplification curves showing the specificity of Cas12a-free LAMP (c) and Cas12a-treated LAMP (d) in the presence of *L. monocytogenes*, *S. aureus*, *Salmonella spp.*, *Escherichia coli*, and *E. sakazakii*. NTC denotes the no-template control.

References

1. Bao Y, Jiang Y, Xiong E, Tian T, Zhang Z, Lv J et al (2020) CUT-LAMP: contamination-free loop-mediated isothermal amplification based on the CRISPR/Cas9 cleavage. ACS Sens 5:1082-1091. https://doi.org/10.1021/acssensors.0c00034.
2. Lin W, Tian T, Jiang Y, Xiong E, Zhu D, Zhou X (2021) A CRISPR/Cas9 eraser strategy for contamination-free PCR end-point detection. Biotechnol Bioeng 118:2053-2066. https://doi.org/10.1002/bit.27718.
3. Hsieh K, Mage P L, Csordas A T, Eisenstein M, Soh H T (2014) Simultaneous elimination of carryover contamination and detection of DNA with uracil-DNA-glycosylase-supplemented loop-mediated isothermal amplification (UDG-LAMP). Chem Commun 50:3747-3749. https://doi.org/10.1039/c4cc00540f.
4. Wang Y, Liu D, Deng J, Wang Y, Xu J, Ye C (2017) Loop-mediated isothermal amplification using self-avoiding molecular recognition systems and antarctic thermal sensitive uracil-DNA-glycosylase for detection of nucleic acid with prevention of carryover contamination. Anal Chim Acta 996:74-87.

https://doi.org/10.1016/j.aca.2017.10.022.
5. Sun K, Liu Y, Tang Q, Ma B, Zhang B, Fu X et al (2025) CADLAB: A contamination-free, argonaute-enhanced, dual detection of plant viruses via lateral flow assay biosensors system. Sens Actuators B 426:137045. https://doi.org/10.1016/j.snb.2024.137045.
6. Zhang T, Zhao W, Zhao W, Si Y, Chen N, Chen X et al (2021) Universally stable and precise CRISPR-LAMP detection platform for precise multiple respiratory tract virus diagnosis including mutant SARS-CoV-2 spike N501Y. Anal Chem 93:16184-16193. https://doi.org/10.1021/acs.analchem.1c04065.
7. Qian C, Wang R, Wu H, Zhang F, Wu J, Wang L (2019) Uracil-mediated new photospacer-adjacent motif of Cas12a to realize visualized DNA detection at the single-copy level free from contamination. Anal Chem 91:11362-11366. https://doi.org/10.1021/acs.analchem.9b02554.
8. Wu F, Lu C, Hu W, Guo X, Chen J, Luo Z (2023) Rapid visual detection of Vibrio parahaemolyticus by combining LAMP-CRISPR/Cas12b with heat-labile uracil-DNA glycosylase to eliminate carry-over contamination. J Zhejiang Univ Sci B 24:749-754. https://doi.org/10.1631/jzus.B2200705.
9. Wen J, Ren L, He Q, Bao J, Zhang X, Pi Z et al (2023) Contamination-free V-shaped ultrafast reaction cascade transferase signal amplification driven CRISPR/Cas12a magnetic relaxation switching biosensor for bacteria detection. Biosens Bioelectron 219:114790. https://doi.org/10.1016/j.bios.2022.114790.
10. Xie Y, Jiang K, Zhang Y, Cao L, Guo X, Shi J et al (2024) Enhanced two-step LAMP-CRISPR assay with an engineered Zst polymerase for contamination-free and ultrasensitive DNA detection. Anal Chem 96:15493-15502. https://doi.org/10.1021/acs.analchem.4c03965.
